# Automated formal synthesis of provably safe digital controllers for continuous plants

**DOI:** 10.1007/s00236-019-00359-1

**Published:** 2019-12-06

**Authors:** Alessandro Abate, Iury Bessa, Lucas Cordeiro, Cristina David, Pascal Kesseli, Daniel Kroening, Elizabeth Polgreen

**Affiliations:** 1 University of California, Berkeley, USA; 2 Department of Computer Science, University of Oxford, Oxford, UK; 3 Department of Electricity, Federal University of Amazonas, Manaus, Brazil; 4 Department of Computer Science, University of Manchester, Manchester, UK; 5 University of Cambridge, Cambridge, UK; 6 DiffBlue Ltd, Oxford, UK

## Abstract

We present a sound and automated approach to synthesizing safe, digital controllers for physical plants represented as time-invariant models. Models are linear differential equations with inputs, evolving over a continuous state space. The synthesis precisely accounts for the effects of finite-precision arithmetic introduced by the controller. The approach uses counterexample-guided inductive synthesis: an inductive generalization phase produces a controller that is known to stabilize the model but that may not be safe for all initial conditions of the model. Safety is then verified via bounded model checking: if the verification step fails, a counterexample is provided to the inductive generalization, and the process further iterates until a safe controller is obtained. We demonstrate the practical value of this approach by automatically synthesizing safe controllers for physical plant models from the digital control literature.

## Introduction

Modern implementations of embedded control systems have proliferated with the availability of low-cost devices that can perform highly non-trivial control tasks, with significant impact in numerous application areas such as process and industrial engineering, high-precision control, automotive and robotics [7, 20]. However, provably correct synthesis of control software for such platforms, needed if certification is in order, is non-trivial even in cases with unsophisticated system dynamics.

In this paper, we examine the case of physical systems (known as ‘plants’ in the control literature) that are mathematically described as linear time invariant (LTI) models, for which the classical synthesis of controllers is well understood. However, the use of digital control architectures adds new challenges caused by artefacts that are specific to digital control, such as the effects of finite-precision arithmetic and quantization errors introduced by A/D and D/A conversion. Given an LTI model, we develop an automatic technique for generating correct-by-design digital controllers that addresses these challenges. Specifically, moving beyond classical literature in digital control [7, 20], we automatically synthesize safe, software-implemented embedded controllers for physical plants.

Our work addresses challenging aspects of control synthesis: we perform automated control synthesis over a model encompassing both a plant exhibiting continuous behavior and a controller operating in discrete time and over a quantized domain. In particular, our model evaluates the effects of the quantizers (A/D and D/A converters), as well as representation errors introduced by the controller working in a finite-precision domain. Our model also accounts for representation errors introduced by our modelling of the plant using finite-precision arithmetic. We reason about specific safety requirements, which are frequently overlooked in conventional feedback control synthesis, but which nevertheless play a key role in safety-critical applications, of clear concern in numerous modern contexts, e.g. autonomy in robotics, avionics, and automotive.

We present a novel approach for the synthesis of digital controllers that makes use of a recently investigated framework known as counterexample-guided inductive synthesis (CEGIS) [23, 43], a technique from formal methods that has recently shown much promise and which we export in this work to a control engineering setup. CEGIS is an iterative process, where each iteration performs inductive generalization based on counterexamples provided by a verification oracle (see Sect. 3.9). The inductive generalization uses information about a limited number of inputs to compute a candidate solution for the entire range of possible inputs.

**Fig. 1:**
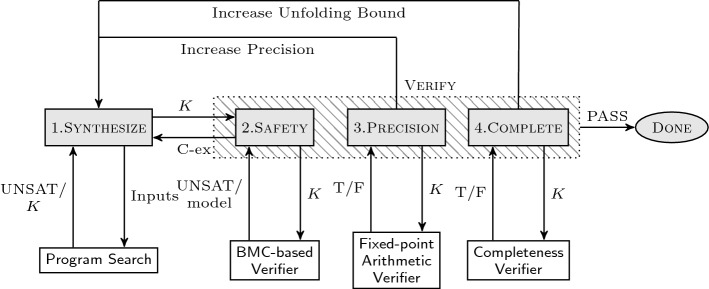
CEGIS with multi-staged verification for digital controller synthesis. We synthesise *K*

**Fig. 2:**
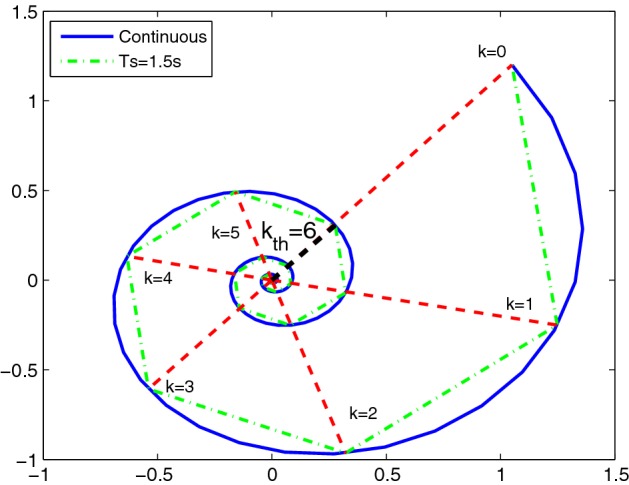
Completeness threshold for multi-staged verification (quantity $T_s$ is the time discretization step)

Our approach uses a multi-staged technique, shown in Fig. 1: it starts by devising a digital controller that stabilizes the plant model while remaining safe for a pre-selected time horizon and a single initial state; then, it verifies an unbounded-time safety requirement by unfolding the model dynamics, considering the full set of initial states, and checking a *completeness threshold* [27]: this is the number of stages required to sufficiently unwind the closed-loop model such that the safety boundaries (which are assumed to be a compact set) are not violated for any larger number of iterations, as illustrated in Fig. 2.

We provide experimental results showing that we are able to efficiently synthesize safe controllers for a set of intricate physical plant models taken from the digital control literature.

In summary this paper, which is an extension of [3], puts forward the following contributions:

1. We automatically generate *correct-by-construction* digital controllers using an inductive synthesis approach (CEGIS). The automatically computed state-feedback controllers guarantee the validity of a given safety specification. This objective, unlike existing methods for controller synthesis that rely on transfer function representations, requires to consider a state-space representation of the physical system. Such a representation ensures the validity of the specification over actual traces of the state-space model, alongside the numerical soundness required by the effects of discretisation and finite-precision errors.
2. We present a novel multi-staged approach to synthesizing digital controllers, using an unfolding of the dynamics up to a completeness threshold. We employ bit-precise bounded model checking to account for fixed-point arithmetic used in the implementation of the digital control algorithm, and interval arithmetic to account for the imprecision in the modelling of the plant.
3. A limitation of the work in [3], which this contribution extends, is its restriction to fixed-point arithmetic, meaning that fixed-point numbers are employed for the representation of both the plant and the controller, as well as for the operations performed by each of them. Conversely, in the current paper we also make use of floating-point arithmetic.

## Related work

### CEGIS

Program synthesis is the problem of computing correct-by-design programs from high-level specifications. Algorithms for this task have made substantial progress over recent years, in particular the architecture known as CEGIS, which is a recent approach to inductive synthesis.

Program synthesizers are an ideal fit for the synthesis of digital controllers, since the semantics of programs precisely capture the effects of finite-precision arithmetic. Surprisingly, the control literature has been oblivious to this relevant connection. A relevant exception is [40], which employs CEGIS on the synthesis of switching controllers for stabilizing continuous-time plants. This work hinges on and is limited by the capacity of the state-of-the-art SMT solvers to reason over linear arithmetic problems. Since this contribution employs finite switching actions for the digital controller, it avoids problems related to finite-precision arithmetic, but potentially suffers from the state-space explosion. Moreover, in [41] the same authors use a CEGIS-based approach for synthesizing continuous-time switching controllers that guarantee *reach-while-stay* properties of closed-loop systems, i.e., properties that specify both a set of goal states and safe states (this specification is also known as constrained reachability). This solution is based on synthesizing control Lyapunov functions for switched systems that yield switching controllers with a guaranteed minimum dwell time in each mode. However, both approaches are unsuitable for the kind of controllers that we seek to synthesize, which are not switching in nature but rather continuous (as further detailed later).

The work in [2] synthesizes stabilizing controllers for continuous plants given as transfer functions, by exploiting bit-accurate verification of software-implemented digital controllers [9]. While this work also uses CEGIS, the approach is restricted to digital controllers for stable closed-loop systems expressed as transfer function models: this results in a static check on their coefficients. By contrast, in the current paper we consider a state-space representation of the physical system, which requires ensuring the specification over actual traces of the model, alongside the numerical soundness required by the effects of discretisation and the errors related to finite-precision arithmetic.

Furthermore, unlike the approach in [2], this work reasons over state-space models. A state-space model has well known advantages over the transfer function representation [20], as it allows synthesis of controllers with guarantees on the internal dynamics, e.g., *safety*. Our work indeed focuses on the safety of internal states (which we assume to fully observe), which is by and large overlooked in the standard (digital) control literature, by default focussed on stability/regulation/tracking properties. Moreover, our work integrates an abstraction-refinement (CEGAR) step inside the main CEGIS loop.

Beyond CEGIS-based architectures, there is an important line of research on provably correct control synthesis for dynamical models, which leverages formal abstractions. The approach underpinning tools such as Pessoa [32], related extensions [4, 30] and applications [51] synthesizes correct-by-design embedded control software by formally abstracting the model to a finite-state machine, and on the formal control synthesis over safety- and reachability-based temporal properties thereon. Whilst in principle this formal abstraction can account for the errors that we can deal with, it is expected to provide a controller of different nature than the one we generate. The obtained finite controller software can then be implemented (refined) over the concrete LTI model. However, relying on state-space discretization, this class of approaches is likely to incur scalability limitations.

### Discretization effects in control design

Recent results in digital control have focused on separate aspects of discretization, e.g. delayed response [17] and finite-precision arithmetic, with the goal either to verify [16] the correctness of the implementation or to optimize [36] its design.

There are two different problems that arise from finite-precision arithmetic in digital controllers. The first is the error caused by the inability to represent the exact state of the physical system, while the second relates to rounding and saturation errors during mathematical operations. In [19], a stability measure based on the error of the digital dynamics ensures that the deviation introduced by finite-precision arithmetic does not lead to instability. Wu et al. [50] uses $\mu$-calculus to synthesise directly a digital controller, so that selected parameters result in stable model dynamics. The analyses in [42, 49] rely on an invariant computation on the discrete dynamics using semi-definite programming (SDP): while the former contribution uses a bounded-input and bounded-output (BIBO) notion of stability, the latter employs Lyapunov-based quadratic invariants. In both cases, the SDP solver uses floating-point arithmetic and soundness is checked by bounding the obtained error. An alternative is [37], where the verification of given control code is performed against a known model by extracting an LTI model of the code via symbolic execution: to account for rounding errors, upper bounds of their values are introduced in the verification phase. The work in [38] introduces invariant sets as a mechanism to bound the effect of quantization error on stabilization. Similarly, [29] evaluates the quantization error dynamics and calculates upper and lower bounds for the possible trajectory of the system, up to a finite time. Considering the problem of multi-modal dynamics, [25] uses numerical optimization techniques to learn optimal switching logic for hybrid systems. The last three approaches can be placed within the research area known as “hybrid systems theory.” The present contribution distances itself from all these cognate results.

A large body of work exists on evaluating fixed-point errors and bridging the gap between real value and fixed-point values in synthesis in other application areas and using other algorithmic techniques: Genetic programming has been used to minimise the error in synthesised fixed-point programs [15]; Smoothed proof search reduces the problem of parameter synthesis under boolean and quantitative objectives to a sequence of optimisation problems [12]; Synthesis of optimal fixed-point implementations of floating-point numerical software can be done using testing and induction [24]. All of these works present potential future applications for CEGIS in synthesising implementations in fixed-point arithmetic.

## Preliminaries

### State-space representation of physical systems

We consider models of physical plants expressed as ordinary differential equations, which we assume to be controllable [5]:

1 $$\begin{aligned} {\dot{x}}(t) = {A}{x}(t)+ {B} {u}(t), \end{aligned}$$

where ${x} \in {\mathbb {R}}^n$, ${u} \in {\mathbb {R}}^p$, ${A} \in {\mathbb {R}}^{n \times n}$, ${B} \in {\mathbb {R}}^{n \times p}$, and $t \in {\mathbb {R}}_0^+$ denotes continuous time. We denote with *x*(0) the model initial condition, which can be non-deterministic. We assume full observability of the states of the model, namely the output states correspond with the model variables, which we can thus fully access.

Equation (1) is discretized in time [33, 48] with constant sampling intervals, each of duration $T_s$ (the sample time), into the difference equation

2 $$\begin{aligned} {x}_{k+1} =&{A}_d {x}_k+ {B}_d {u}_k, \end{aligned}$$

where ${A}_d=e^{{A}T_s}$ and ${B}_d = \int _{t = 0}^{T_s} e^{{A} t} dt\ {B}$, and where $k \in {\mathbb {N}}$ is a discrete counter and ${x}_{0}={x}(0)$ denotes the initial state. We assume that specifications concern the model in (2), and we plan to devise controllers ${u}_k$ to meet them. (The more general problem of synthesising controllers *u*(*t*) for (1) fall outside the scope of the present work.)

### Digital control synthesis

**Fig. 3:**
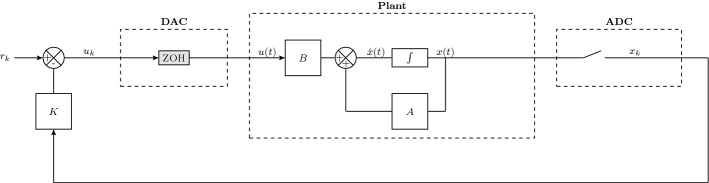
Closed-loop digital control setup, comprising an analogue model of the underlying real system, alongside a digital controller

Models (1) and (2) depend on external non-determinism in the form of input signals *u*(*t*) and $u_k$, respectively. Feedback architectures can be employed to manipulate properties and behaviors of the plant: we are interested in the synthesis of digital feedback controllers $u_k$, as in Fig. 3, as practically implemented on field-programmable gate arrays or on digital signal processors, and as classically studied in [7].

Recall that the states of the model are fully accessible (namely, observable). We consider state feedback control architectures, where $u_k$ (notice we work with the discretized signal) is $u_k = r_{k} - K x_k$. Here $K \in {\mathbb {R}}^{p \times n}$ is a state-feedback gain matrix, and $r_{k}$ is a reference signal (again digital). We will assume $r_k=0$ (meaning that the reference signal is just a zero signal), thus obtaining the closed-loop model ${x}_{k+1}=({A}_d-{B}_d{K}){x}_k$ with the origin as its equilibrium point.

The gain matrix *K* can be set so that the closed-loop discrete dynamics are shaped as desired, for instance according to a specific stability goal or around a dynamical behavior of interest [7]. As argued later in this work, we will target a less standard objective, namely a quantitative safety requirement defined over a convex set around the origin, which opens up to more complex specifications [8, 45]. This is not typical in the digital control literature. We will further precisely account for the digital nature of the controller, which manipulates quantised signals as discrete quantities represented with finite precision. The new approach is fully automated and leverages an approach based on CEGIS, to be introduced shortly.

### Stability of closed-loop models

In this work we employ the notion of asymptotic stability within the CEGIS loop, as a means for reducing the search space of possible safe controllers, where the notion of a safe controller is defined in the following section. As discussed later, for linear models a safe controller is necessarily asymptotically stable, although the reverse is not true. Qualitatively, (local) asymptotic stability is a property denoting the convergence of the model executions towards an equilibrium point, starting from any states in a neighborhood of the point. In the case of linear systems considered with a zero reference signal (as assumed above), the equilibrium point of interest is the origin (see Fig. 2 for the portrait of an asymptotically stable execution, converging to the origin).

Of interest for this work, it can be shown that a discrete-time LTI model is asymptotically stable if all the roots of its characteristic polynomial (i.e., the eigenvalues of the closed-loop matrix $A_d - B_d K$) are inside the unit circle on the complex plane, i.e., if their absolute values are strictly less than one [7]. Whilst this simple sufficient condition can be either generalized or strengthen to be necessary, this is not necessary in the context of this work. What is technically key is that in this paper we shall express this asymptotic stability as a specification $\phi _ stability$, and encode it in terms of a check known as *Jury’s criterion* [18]: this is an easy algebraic formula to check the entries of matrix *K*, so that the closed-loop dynamics are shaped as desired. We refer the interested reader to [18] for further details about Jury’s criterion, which we omit for the sake of space and since it is a standard result in the control literature that we just leverage for our overall approach.

### Safety of closed-loop models

We are not limited to the synthesis of digital stabilizing controllers—a well known task in the literature on digital control systems—but target safety requirements with an overall approach that is sound and automated. More specifically, we require that the closed-loop model meets a given safety specification that is characterised by a given set around the origin. A safety specification gives rise to a requirement on the states of the model, namely that they remain within the safe set at all times (that is, over an infinite number of time steps). So the feedback controller (namely the choice of the gain matrix *K*) must ensure that the state never violates the requirement. Note that an asymptotically stable, closed-loop system is not necessarily a safe system: indeed, the state values may leave the safe part of the state space while they converge to the equilibrium, which is typical in the case of oscillatory dynamics. In this work, the safety property is expressed as:

3 $$\begin{aligned} \phi _ safety = \left\{ \forall k\ge 0,\, \bigwedge _{i=1}^{n}{\underline{x_{i}} \le x_{i,k} \le \overline{x_{i}}}\right\} , \end{aligned}$$

where $\underline{x_{i}}$ and $\overline{x_{i}}$ are lower and upper bounds for the *i*th coordinate $x_{i}$ of state $x\in {\mathbb {R}}^n$ at the *k*th instant, respectively. This requires that the states will always be within an *n*-dimensional hyper-box.

Beyond the main requirement on safety, it is practically relevant to consider the constraints $\phi _ input$ on the input signal $u_{k}$ and $\phi _ init$ on the initial states $x_0$, which we assume have given bounds: $\phi _ input = \{\forall k \ge 0,\bigwedge _{i=1}^{p} {\underline{u}} \le u_{i} \le {\overline{u}}\}$, and $\phi _ init = \{ \bigwedge _{i=1}^{n} \underline{x_{i,0}} \le x_{i,0} \le \overline{x_{i,0}}\}$. The former constraint expresses that the control input, possibly shaped via state-feedback, might saturate in view of physical constraints. Notice that we will assume that the set of initial states lies within the safe set, since the contrary leads to dynamics that are trivially unsafe. Furthermore, whilst the problem can handle general hyper-boxes, we will in practice work with sets that contain the origin, particularly in view of the choice of the reference signal $r_k = 0$.

### Bounded model checking

We employ the bounded model checking software tool CBMC [28] to model the controller behavior on digital signals with finite-bit precision. We express the controller semantics as C programs, which is CBMC’s preferred input language. CBMC symbolically executes every instruction in an input program by translating it to a Boolean satisfiability problem, which is satisfiable iff a certain property about the program holds. CBMC’s API allows to specify these properties in the form of assertions in the input program: we set these properties to express that our controllers generate (asymptotically stable and) safe dynamics.

### Semantics of finite-precision arithmetic

A key contribution of this work is that it precisely replicates the finite-precision arithmetic within the digital controller it synthesizes, thus guaranteeing that controllers implemented with finite-precision arithmetic are safe. The specific components of the model we are concerned with, shown in Fig. 3, are the ADC, the digital controller and the DAC. Details of how we model the behaviour of the finite-precision arithmetic in these components are in Sect. 5. We must model the semantics precisely, in order to capture the full behaviour of the model. More specifically, we encompass the following features:

- The ADC converts the analog signal *x*(*t*) into a digital signal $x_k$, which is then fed into the controller, converting a real value to a finite-precision value.
- The controller block performs arithmetic at finite precision. We assume the ADC represents numbers with at least the same precision as the controller, and thus focus on the precision as limited by the controller. This is a reasonable assumption based on commonly available hardware.
- The DAC converts finite-precision values back to real values. We assume that the input to the DAC has the same precision as the output of the controller. It would, however, be straightforward to account for a DAC or ADC of different precision than the controller in our algorithm, if necessary.

### Soundness of modelling

In addition to precisely replicating the finite-precision arithmetic of the digital controller, we must consider that our *model* itself in (2) employs finite-precision arithmetic to represent the behaviour of the real system. In order to guarantee soundness, we therefore encompass the error that is due to modelling (as opposed to the nature of the digital controller): the representations used in the plant model and its arithmetic operations are carried out at finite precision. More specifically:

- We account for the error introduced by finite-precision arithmetic applied over the model variables $x_k$ and $u_k$, which are actually continuous quantities. We guarantee that the precision we use to represent the model variables is at least as precise as the precision used in the digital controller, and we use interval arithmetic to bound the incurred errors, as further detailed in Sect. 5.

### Notation for fixed- and floating-point precision

In this paper we will use ${\mathcal {F}}_{\langle I,F \rangle }(x)$ to denote a real number *x* expressed with a fixed-point precision, using *I* bits to represent the integer part of the number and *F* bits to represent its decimal part. In particular, ${\mathcal {F}}_{\langle I_c,F_c \rangle }(x)$ denotes a real number *x* represented at the fixed-point precision of the controller, and ${\mathcal {F}}_{\langle I_p,F_p \rangle }(x)$ denotes a real number *x* represented at the fixed-point precision of the plant model. $I_c$ and $F_c$ are determined by the controller. We pick $I_p$ and $F_p$ for our synthesis such that $I_p \ge I_c$ and $F_p \ge F_c$, so that our controller can be represented accurately in the model domain. Thus any mathematical operations in our modelled digital controller will be in the range of ${\mathcal {F}}_{\langle I_c,F_c \rangle }$, and all other calculations in our model will be carried out in the range of ${\mathcal {F}}_{\langle I_p,F_p \rangle }$.

We further employ ${\mathcal {F}}_{\langle E,M \rangle }(x)$ to denote a real number *x* represented in a floating-point domain, with *E* bits representing the exponent part and *M* bits representing the mantissa part. In particular, we use ${\mathcal {F}}_{\langle E_c,M_c \rangle }(x)$ to denote a real number represented at the floating-point precision of the controller, whereas ${\mathcal {F}}_{\langle E_p,M_p \rangle }(x)$ denotes a real number represented at the floating-point precision of the plant model.

**Fig. 4:**
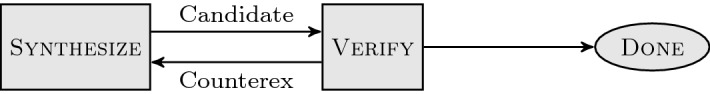
The general CEGIS framework

### Counterexample-guided inductive synthesis (CEGIS)

In this section, we give a brief description of the CEGIS framework [23, 43], which is illustrated in Fig. 4. CEGIS has been recently developed for the automated synthesis of software programs, and its setup is naturally that of mathematical logic. We consider an input specification of the form

$$\begin{aligned} \exists P .\, \forall a.\, \phi (a, P), \end{aligned}$$

where *P* ranges over functions (where a function is represented by the program computing it), *a* ranges over ground terms, and $\phi$ is a quantifier-free logical formula. We interpret the ground terms over some domain ${\mathcal {D}}$. This is a design problem, where the objective is to synthesise a valid *P* that satisfies $\phi$ over all the *a* terms.

CEGIS breaks down this generally hard synthesis problem into two easier parts: an inductive synthesis phase (denoted by Synthesize in Fig. 4) and a verification phase (denoted by Verify in Fig. 4), which interacts via a finite set of tests inputs that is updated incrementally. Given the specification $\phi$, the inductive synthesis procedure tries to find an existential witness *P* satisfying the specification $\phi (a, P)$ for all *a* in inputs (as opposed to all $a \in {\mathcal {D}}$). If the synthesis phase succeeds in finding a witness *P*, this witness is a candidate solution to the full synthesis formula. We pass this candidate solution to the verification phase, which checks whether it is a full solution (i.e., *P* satisfies the specification $\phi (a, P)$ for all $a\in {\mathcal {D}}$). If this is the case, then the algorithm terminates. Otherwise, additional information is provided to the inductive synthesis phase in the form of a new counterexample that is added to the inputs set and the loop iterates again. If the solution space is finite then the CEGIS loop is guaranteed to terminate by either finding a solution or showing that no solution exists.

In the context of the formal synthesis of safe controllers, of interest for this work, the set of possible inputs corresponds to the set of possible initial states and the candidate program *P* is a candidate controller *K*. The synthesis block generates a candidate controller that works for a subset of the possible initial states, and the verifier checks whether the controller works for all possible initial states.

## Formal specification of stability on a model

Since we are interested in capturing safety [as encoded in Eq. (3) in Sect. 3.4], we use a stability specification to narrow the search space of possible controllers, as detailed in Sect. 4.1. Essentially, we employ stability as a precursor to safety.

### Jury’s stability criterion

There are a number of well known procedures to perform stability analysis of dynamical models [5]. Here we select the classical *Jury’s stability criterion* [7], in view of its efficiency and ease of integration within our implementation. This method checks the stability of a model working over the complex domain of its characteristic polynomial *S*(*z*), considered in its general form as

$$\begin{aligned} S(z) = a_0z^N+a_1z^{N-1}+\cdots +a_{N-1}z+a_N,{\quad } a_0\ne 0. \end{aligned}$$

A standard result in Control theory [5] states that this polynomial can be obtained as a function of the state-space matrices $A_d,B_d$, and in particular its order *N* corresponds to the dimensions of the state variables (above, *n*). A sufficient condition for asymptotic stability of the closed-loop LTI model [7] is when all the roots of its characteristic polynomial *S*(*z*) (which correspond to the eigenvalues of the matrix $A_d-B_dK$) are inside the unit circle in the complex plane, i.e., when the absolute values of the roots are less than one.

Skipping the full algebraic derivation for the sake of space (this can be found in [7]), the following matrix *M* with dimension $(2N-2)\times N$ and elements $m_{(\cdot ),(\cdot )}$ is built from the coefficients of *S*(*z*) as:

$$\begin{aligned} M=\left( \begin{array}{c} V^{(0)}\\ V^{(1)}\\ \vdots \\ V^{(N-2)} \end{array} \right) , \end{aligned}$$

where $V^{(k)} = [v^{(k)}_{ij} ]_{2\times N}$ is such that:

$$\begin{aligned} v_{ij}^{(0)}= & {} \left\{ \begin{array}{l@{\quad }l} a_{j-1}, &{} \text{ if }~i=1\\ v_{(1)(N-j+1)}^{0},&{}\text{ if }~i=2 \end{array} \right. \\ v_{ij}^{(k)}= & {} \left\{ \begin{array}{l@{\quad }l} 0,&{}\text{ if }~j>n-k\\ v_{1j}^{(k-1)}-v_{2j}^{(k-1)} . \frac{v_{11}^{(k-1)}}{v_{21}^{(k-1)}}, &{} \text{ if }~j\le n-k ~\text{ and }~i=1\\ v_{(1)(N-j+1)}^{k},&{} \text{ if }~j\le n-k ~\text{ and }~i=2\\ \end{array} \right. \end{aligned}$$

and where $k \in {\mathbb {Z}}$ is such that $0< k < N - 2$.

We have that *S*(*z*) is the characteristic polynomial of an asymptotically stable system if and only if the following four conditions $R_i, i = 1,2,3,4,$ hold [7]: $R_1: S(1) > 0$; $R_2: (-1)^N S(-1) > 0$; $R_3: |a_0| < a_N$; $R_4: m_{11}> 0 \wedge m_{31}>0 \wedge m_{51}>0 \wedge \ldots \wedge m_{(2N{-}3)(1)}>0$, where $m_{ij}$ denotes the element in position (*i*, *j*) of the matrix *M*, as defined previously.

Finally, the asymptotic stability property is finally encoded by a constraint expressed as the following formula: $\phi _ stability = \{R_1 \wedge R_2 \wedge R_3 \wedge R_4\}.$

## Numerical representation and soundness

As discussed in Sect. 3.6, the considered models must account for the semantics of finite-precision arithmetic, deriving from several sources: we formally bound the numerical error introduced by the finite-precision representation of the plant (and its operations), and precisely model the behaviour introduced by the ADC/DAC conversions, as well as the behaviour of the limited-precision arithmetic used in the controller.

Technically, we employ interval arithmetic to bound the error introduced by the finite-precision plant model, and we use bounded model checking to precisely model the semantics of finite-precision arithmetic, as introduced by the ADC/DAC blocks and by the finite-precision controller.

### Bit-precise bounded model checking

As described in Sect. 3.5, we use CBMC, a bit-precise bounded model checker, to synthesise and verify candidate controllers. CBMC manipulates precisely the fixed- or floating-point arithmetic used in the controller, as well as the ADC/DAC conversions, according to the IEEE standards.

### Interval arithmetic for errors in numerical representations

We use finite-precision arithmetic to model the plant. This is an approximation that speeds up each CEGIS iteration, however it necessitates a further stage where we verify that the errors introduced by the approximation have not resulted in a controller that is unsafe when executed on a model expressed over real numbers. In this stage, we represent the plant model using double-precision floating-point numbers and we use the Boost interval arithmetic library [11] to bound the error in this representation. We employ a compositional numerical library to model the fixed-point arithmetic for the controller^1^ within double-precision floating-point numbers. We check that the controller is safe starting from each vertex of the set of initial states, and show that this is sufficient to prove safety from any state in this set (see Theorem 1).

We describe here the mathematics behind bounding the errors on the double-precision floating-point numbers. Recall we use ${\mathcal {F}}_{\langle E,M \rangle }(x)$ denote a real number *x* represented in a floating-point domain, with *E* bits representing the exponent part, and *M* bits representing the mantissa. In general the representation of a real number using the floating-point domain introduces an error, for which an upper bound can be given [10]. For each number *x* represented in the floating-point domain as ${\mathcal {F}}_{\langle E,M \rangle }(x)$, we store an interval that encompasses this error. Further mathematical operations performed at the precision ${\mathcal {F}}_{\langle E,M \rangle }(x)$ will propagate this error, leading to further errors for which bounds can be derived [10].

The fixed-point arithmetic of the digital controller is performed on the upper and lower bound of the intervals from above independently, and the upper and lower bound of the result is taken as the interval result. For example, consider the conversion from the model precision to controller precision performed by the ADC on a single state value. The state value is represented as an interval $\{x.high, x.low\}$, and the result of the conversion is an interval where the upper bound is the conversion of *x*.*high* and the lower bound is the conversion of *x*.*low*. Since the precision of the floating-point domain is greater than the precision of the controller, this is guaranteed to bound the real behaviour of the controller.

### Effect of finite-precision arithmetic on safety specification and on stability

In this section we will quantify how the finite-precision arithmetic in a digital controller affects the safety and stability properties of an LTI model.

#### Safety of closed-loop models with finite-precision controller error

Let us first consider the effect of the quantization errors on safety. Within the controller, state values are manipulated at low precision, by means of the vector multiplication *Kx*. The inputs are thus computed using the following equation:

$$\begin{aligned} u_{k}&=-({\mathcal {F}}_{\langle I_c,F_c \rangle }(K)\cdot {\mathcal {F}}_{\langle I_c,F_c \rangle }(x_{k})). \end{aligned}$$

This induces two types of errors, as detailed above: first, the truncation error due to the representation of $x_k$ as ${\mathcal {F}}_{\langle I_c,F_c \rangle }(x_{k})$; and second, the rounding error introduced by the multiplication operation. Recall that both these errors are modelled precisely by bounded model checking.

An additional error is due to the representation of the plant dynamics, namely

$$\begin{aligned} x_{k+1} ={\mathcal {F}}_{\langle I_p,F_p \rangle }(A_d) {\mathcal {F}}_{\langle I_p,F_p \rangle }(x_{k}) + {\mathcal {F}}_{\langle I_p,F_p \rangle }(B_d){\mathcal {F}}_{\langle I_p,F_p \rangle }(u_{k}). \end{aligned}$$

We encompass this error using interval arithmetic [34] in the precision check shown in Fig. 1 and detailed in the previous section.

#### Stability of closed-loop models with fixed-point controller error

The validity of Jury’s criterion [18] relies on the representation of the closed-loop dynamics $x_{k+1} = (A_d - B_dK) x_k$ at infinite precision. When we employ a digital controller with fixed-point arithmetic, the operation above can be expressed as follows, where we use ${\mathcal {F}}_{\langle I_c,F_c \rangle }$ preceding a variable to indicate that variable is converted into the fixed-point precision given by ${\mathcal {F}}_{\langle I_c,F_c \rangle }$:

$$\begin{aligned} x_{k+1}&= A_d \cdot x_{k} -B_d({\mathcal {F}}_{\langle I_c,F_c \rangle }(K)\cdot {\mathcal {F}}_{\langle I_c,F_c \rangle }(x_{k})). \end{aligned}$$

This translates to

$$\begin{aligned} x_{k+1}&= (A_d - B_dK) \cdot x_k + B_dK\delta , \end{aligned}$$

where $\delta$ is the maximum error that can be introduced by the digital controller in one step, i.e., by reading the states values once and multiplying by *K* once. We derive the closed form expression for $x_n$ recursively, as follows:

$$\begin{aligned} x_{1}&= (A_d - B_dK)x_0 + B_dK\delta \\ x_{2}&=(A_d - B_dK)^2x_0 + (A_d - B_dK)B_dK\delta + B_dK\delta \\ x_{n}&= (A_d - B_dK)^nx_0 + (A_d - B_dK)^{n-1}B_dK\delta +\\&\quad \cdots + (A_d - B_dK)^1B_dK \delta + B_dK\delta \\&= (A_d - B_dK)^nx_0 + \sum _{i=0}^{n-1}(A_d - B_dK)^iB_dk\delta . \end{aligned}$$

Recall that a closed-loop asymptotically stable system will converge to the origin. We know that the original system with an infinite-precision controller is stable, because we have synthesized it to meet Jury’s criterion. Hence, $(A_d - B_dK)^n x_0$ must converge to zero as $n \uparrow \infty$. Furthermore, the power series of a square matrix *T* converges [22] iff the eigenvalues of the matrix are less than 1, and the limit results in $\sum _{i=0}^{\infty }T^i = (I - T)^{-1}$, where *I* is the identity matrix. Thus, the closed-loop model converges to the value

$$\begin{aligned} 0 + (I - A_d + B_dK)^{-1}B_dk\delta \,. \end{aligned}$$

As a result, if the value $(I - A_d + B_dK)^{-1}B_dk\delta$ is within the safe set of states given by the safety specification, then the synthesized fixed-point controller results in a safe closed-loop model. The convergence to a finite value, however, will not make it asymptotically stable. Since in this paper we require stability only as a precursor to safety, it is thus sufficient to check that the perturbed model converges to a neighborhood of the equilibrium within the safe set.

A similar argument can be made for floating-point arithmetic. In conclusion, we can thus disregard these steady-state errors (caused by finite-precision arithmetic) when stability is ensured by synthesis, and then verify safety accounting for the finite-precision errors.

## Synthesis of digital controllers with CEGIS

In this section we discuss the CEGIS procedure that is used to synthesise safe digital controllers, accounting for the precision issues detailed in the previous sections. We employ a multi-stage approach that unwinds the dynamics of the model up to a completeness threshold, encompassing finite-precision arithmetic using bit-precise bounded model checking, and then verifying soundness of the resulting controller using interval arithmetic.

An overview of the algorithm for controller synthesis is given in Fig. 1. One important point is that we formally synthesize a controller over a finite number (*k*) of time steps (i.e., it is multi-stage). We then compute a completeness threshold ${\overline{k}}$ [27] for this controller, and verify the correct behaviour for ${\overline{k}}$ time steps. As we will later argue, ${\overline{k}}$ is the number of iterations required to sufficiently unwind the dynamics of the closed-loop state-space model, ensuring that the safety boundaries are not violated for any other $k{>}{\overline{k}}$.

Next, with reference to the CEGIS scheme in Fig. 4, we describe in detail the different phases in Fig. 1 (shaded blocks 1 to 4).

### **synthesize** block

The inductive synthesis phase (synthesize) uses BMC to compute a candidate solution *K* that satisfies both the stability requirement and the safety specification, within a finite-precision model. In order to synthesize a controller that satisfies the stability requirement, we need the characteristic polynomial of the closed-loop model to satisfy Jury’s criterion [18] (see Sect. 4.1).

We fix an index *k*, and we synthesize a safe controller by unfolding the transition system (i.e., the closed-loop model) *k* steps and by selecting a controller *K* and a single initial state, such that the states at each step do not violate the safety requirement (see Sect. 3.4). That is, we ask the bounded model checker [14] if there exists a *K* that is safe for at least one $x_0$ in our set of all possible initial states, and a given fixed-point precision for the controller, and where the input signal remains within the specified bounds. The bounded model checker selects the controller *K* values using a satisfiability solver, i.e., by constraint solving. As such, we do not need to consider traditional control approaches such as pole placement. This approach is sound, i.e., the controller produced is guaranteed to be safe, if the current *k* is greater than the completeness threshold (see later step). We also assume some finite precision for the model and a given time discretisation, as described in Sect. 3.1. We use a fixed-point precision in this description, given by $\langle I_p,F_p\rangle$, but if we are considering a floating-point controller we will instead model the plant with a floating-point precision, $\langle E_p,M_p\rangle$. The checks that these assumptions hold are performed by the subsequent verify stages.
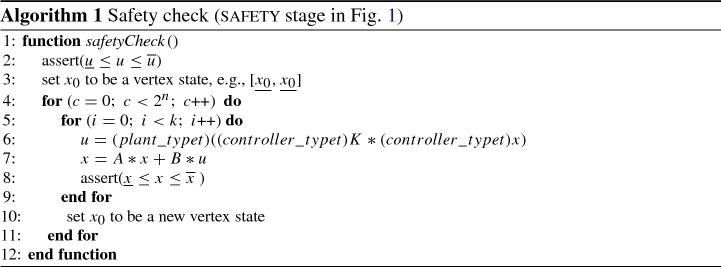


### **safety** block

The first verify stage, safety is shown in Algorithm 1. The algorithm checks that the candidate solution *K*, which we have synthesized to be safe for at least one initial state, is safe for *all* possible initial states, i.e., it does not reach the (complement) unsafe set within *k* steps. After unfolding the transition system corresponding to the previously synthesized controller *k* steps, we check that the safety specification holds for any initial state.

We use $(controller\_typet)$ to denote a cast or conversion to the controller precision, and $(plant\_typet)$ to denote a cast or conversion to the model precision. A *vertex state* is defined as a state where all values are either equal to the upper or lower bound of the states in the initial set. It is sufficient to verify that the controller is safe for all vertex states, as shown next.

#### Theorem 1

Assuming that the initial set is included within the safe set, if a controller is safe for each of the corner cases of the hyper-box of allowed initial states, i.e., the vertex states, then it is safe for any initial state in the hyper-box.

#### Proof

Consider the set of initial states, $X_0$, which we note is convex since it is a hyper-box. Name $v_i$ its vertices, where $i=1,\ldots , 2^n$. Thus any point $x \in X_0$ can be expressed by convexity as $x = \sum _{i=1}^{2^n} \alpha _i v_i$, where $\sum _{i=1}^{2^n} \alpha _i =1$. Then if $x_0=x$, we obtain

$$\begin{aligned} x_k&= (A_d - B_d K)^k x = (A_d - B_d K)^k \sum _{i=1}^{2^n} \alpha _i v_i \\&= \sum _{i=1}^{2^n} \alpha _i (A_d - B_d K)^k v_i = \sum _{i=1}^{2^n} \alpha _i x_k^i, \end{aligned}$$

where $x_k^i$ denotes the trajectories obtained from the single vertex $v_i$. We conclude that any *k*-step trajectory is encompassed, within a convex set, by those generated from the vertices. Recall that we have assumed that the initial set lies within the safe set, and that both are (convex) hyper-boxes. The conclusion follows. $\square$

Summarising, we only need to check $2^n$ initial states, where *n* is the dimension of the state space (number of continuous variables). Whilst this full check on all vertices might not be necessary in general (it might be simplified in special cases or under special conditions), it does not represent the bottleneck of our overall method, so we can safely rely on its current form.

### **precision** block

The second verify stage, precision, restores soundness with respect to the plant model precision by using interval arithmetic [34] to validate the operations performed by the previous stage.

### **complete** block

The third and last verify stage, complete, checks that the current *k* is large enough to ensure safety for any further time steps. Here, we compute the completeness threshold ${\overline{k}}$ for the current candidate controller *K* and check that $k{\ge }{\overline{k}}$. This is done by computing the number of time steps required for the states to have completed a 360° circle, as illustrated in Fig. 2.

#### Theorem 2

There exists a finite ${\overline{k}}$ such that it is sufficient to unwind the closed-loop state-space model up to ${\overline{k}}$ in order to ensure that $\phi _ safety$ holds.

#### Proof

An asymptotically stable model is known to have converging dynamics. Assume that the eigenvalues of the closed-loop matrix are not repeated: this is sensible assumption to raise, since the eigenvalues are selected by the user. The distance of the trajectory from the reference point (which, for linear models, is the origin) decreases over time within subspaces related to real-valued eigenvalues (say, $\theta < 0$): this can be shown considering the exponential $e^{\theta t} x_0$ ($x_0$ being the initial condition), which envelops the model dynamics within subspaces and is monotonically decreasing. However, this monotonic decrease cannot be ensured in general when dealing with complex eigenvalues. In this second instance, consider the closed-loop matrix that updates the states at each discrete time step, and select the eigenvalue $\vartheta$ with the smallest (non-zero) imaginary part. Between any pair of consecutive time steps $k\,T_s$ and $(k+1)\,T_s$, the dynamics projected on the corresponding eigenspace rotate of $\vartheta T_s$ radians. Thus, taking ${\overline{k}}$ as the ceiling of $\frac{2\pi }{\vartheta T_s}$, after $k{\ge }{\overline{k}}$ steps the model trajectory has completed a full rotation within the relevant eigenspace: this results in a point closer to the origin, as shown in Fig. 2. The synthesized ${\overline{k}}$ is thus a completeness threshold: indeed, since we have selected $\vartheta$ to be the complex eigenvalue with smallest imaginary part, any other pair of complex eigenvalues will correspond to dynamics that rotate faster within the corresponding eigenspace. Hence, ${\overline{k}}$ will again represent a global completeness threshold. $\square$

## DSSynth: a software tool for automated digital controller synthesis over physical plants

**Fig. 5:**
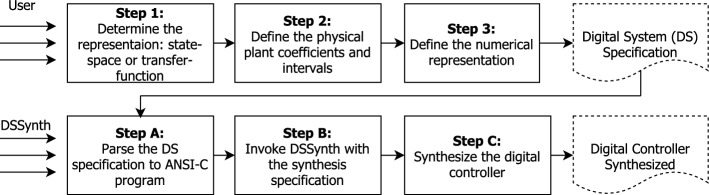
The digital-system synthesizer (DSSynth) tool—distinct phases of the controller synthesis

The implementation of the proposed methodology for synthesis of digital controls for physical plants is based on the digital-system synthesizer (DSSynth) tool [1], which can be split into two main stages: manual and automated, as illustrated in Fig. 5 and detailed next.

The first stage comprises the following steps. In Step 1, the user selects the system representation, which can be a transfer function or a state-space model (we focus on the latter in this article). In Step 2, the plant [e.g., in the form of Eq. (1)] is provided [7]. Finally, in Step 3, the numerical representation for the digital controller implementation must be set by the user: this is the finite-precision arithmetic that defines the number of bits of integer and fractional parts when using fixed-point arithmetic, or half- and single-precision when using floating-point arithmetic. The user also specifies the input range.

In the second stage, the automated synthesis process starts with Step A, where the DSSynth translates the model specification into an ANSI-C program. In Step B, the discussed CEGIS engine is invoked, in order to synthesize the digital controller w.r.t. the specification given on the model. Finally, in Step C, the synthesized digital controller is generated. The output of DSSynth is the closed-loop model with the synthesized digital controller, which is represented either as a transfer function or in state-space form (in this article we consider the latter case). The synthesis is considered to be *successful* if a digital controller is correctly synthesized with respect to the effects of finite-precision arithmetic within a time-out set to 5 h.

The CEGIS engine is implemented as an integrated module within the C bounded model checker (CBMC) [14]. CBMC transforms the ANSI-C representation of the closed-loop control model model into its internal representation (IR). We instrument this IR for each synthesis or verification scenario accordingly, and use CBMC as an oracle to answer our queries. CBMC itself relies on an underlying SAT/SMT solver to address verification checks. We model the effects of finite-precision arithmetic explicitly using CBMC’s nondeterminism API (e.g., nondet, CPROVER_assume intrinsic functions).

## Experimental evaluation

Our benchmark suite consists of 19 case studies extracted from the control literature [13, 20, 21, 21, 26, 35, 44, 46, 47]. These case studies comprise control models, which we represent in state space form as in Eq. (1). The models are time discretized, with sampling times ranging from 1 to 0.0001 s. CEGIS initially starts with the coarsest discretisation (i.e., 1s sampling time), and if it fails to find a controller, it reduces the sampling time. The initial states are bounded between 0.5 and $-0.5$ for all states, and the safety specification requires that the states remain between $-1.5$ and 1.5 (as remarked above, the initial set lies within the safe set and contains the origin). The input bounds are selected individually for each benchmark.

### Description of the control benchmarks

The *bioreactor* benchmark is a linear model of the cell mass concentration controlled through the dilution rate of a bioreactor [39]. The *Chen* benchmark correspond to a higher-order control system model employed as a case study for model-order reduction techniques [13]. The benchmarks *Cruise 1* [20] and *Cruise 2* [5] deal with automotive cruise control models, where the control is used to maintain the speed of an automobile at a constant value (after tracking a desired speed reference), and compensating disturbances and uncertainties. The models *cstr* and *cstrtmp* describe the pH dynamics of a reaction of an aqueous solution of sodium acetate with hydrochloric acid [46] and the temperature of a reaction [6] in a tank reactor. The *DC motor* plant describes the velocity dynamics of a direct-current electrical machine. The *helicopter* benchmark plant describes the transitional and rotational dynamics of a coaxial helicopter. The *inverted pendulum* benchmark describes a model for the cart position and for the angle of an inverted pendulum placed on the cart, which moves over a track by means of a DC motor. The *magnetic pointer* benchmark describes a magnetic pointer, whose angular position dynamics is controlled by a magnetic field. The *magsuspension* describes the dynamics of a magnetic car suspension system. The *pendulum* plant consists of a swinging point mass suspended from a frictionless pivot by means of a rod with negligible mass. The *Regulator* consists of a linear model of a synchronous electrical machine [26]. The *satellite attitude control system* plant describes the dynamics of a satellite attitude, *i.e.*, the orientation angles. An attitude control system must maintain the desired orientation of a satellite with respect to an inertial frame. The *springer-mass damper system* plant is a standard model for the dynamics of several mechanical systems. The *steam drum* benchmark describes a linear model for the level dynamics of a boiler steam drum [31]. The *suspension* models the single-wheel suspension system of a car, namely the relative motion dynamics of a mass-spring-damper model, which connects the car to one of its wheels. The *tapedriver* benchmark models a computer tape driver, that is a computer storage device used to read and write data on magnetic tapes. The *USCG cutter tampa heading angle* plant describes the heading angle dynamics of a Coast Guard cutter.

### Objectives

Using the state-space models described in Sect. 8.1, the evaluation study has the following overall experimental goal:

- Show that the multi-staged CEGIS approach is able to generate finite-precision digital controllers using fixed-point and floating-point arithmetic in a reasonable amount of time.

### Results

We provide the results in Table 1, where: *Benchmark* is the name of the corresponding benchmark; *Order* is the number of continuous variables of the model; ${\mathcal {F}}_{\langle I_p,F_p \rangle }$, where $I_p$ and $F_p$ indicate the integer and fractional parts, and ${\mathcal {F}}_{\langle E_p,M_p \rangle }$, where $E_p$ and $M_p$ indicate the exponent and mantissa part, are the fixed- and floating-point precisions used to model the plant, respectively; and *Time* is the total time required to synthesize a controller for the given model. Time-outs are indicated by ✗, where the time-out used is 5 h. The precision for the controller, ${\mathcal {F}}_{\langle I_c,F_c \rangle }$, is chosen to be $I_c = 8$, $F_c = 8$ for fixed-point, whereas ${\mathcal {F}}_{\langle E_c, M_c \rangle }$ is chosen to be $W_c = 6$ and $F_c = 10$ for half-precision floating-point format.

We separate our evaluation into two sets of results: fixed- and floating-point. We present a selection of case-studies that we are able to solve either for floating-point or fixed-point. Given a time discretisation, the floating-point controllers in general take longer to synthesise than the fixed-point ones. However, the fixed-point algorithm is often forced to try more time discretisations, because the fixed-point controller lacks the precision to control the system at the coarser time discretisation.

**Table 1:** Synthesis times for fixed- and floating-point controllers

Benchmark	Order	${\mathcal {F}}_{\langle I_p,F_p \rangle }$	Time (s)	k	${\mathcal {F}}_{\langle E_p, M_p \rangle }$	Time (s)	k
Bioreact	2	8,8	15.35	4	10,6	23.76	2
Chen	3	8,8	11.24	0	10,6	14.25	0
Cruise	1	8,8	11.03	0	10,6	11.17	0
Cruise 2	1	8,8	9.93	0	10,6	10.54	0
Cst	3	12,12	90.03	2	10,6	321.12	2
Cstrtmp	2	8,8	18.56	2	10,6	16.99	2
DC motor	2	8,8	10.34	0	10,6	12.32	0
Helicopter	3	16,16	1116.08	2	10,6	168.43	38
Inverted pendulum	2	12,12	16.01	2	10,6	18.73	0
Magnetic pointer	3	12,12	1071.02	10	10,6	207.60	9
Magnetic suspension	3	20,20	56.9	2	10,6	998.3	6
Pendulum	2	8,8	11.74	0	10,6	13.69	0
Regulator	5		✗		10,16	190.28	2
Satellite	2	8,8	13.91	3	10,6	16.92	7
Spring-mass-damper	2	12,12	16.09	0	10,6	23.21	4
Steam drum	3		✗		10,16	21.16	4
Supension	4	8,8	12.40	5	10,6	17.03	5
Tape driver	3	8,8	12.18	0	10,6	14.34	0
USCG tampa	3	12,12	1143.35	10	10,6	210.70	9

The mean run-time for the successful benchmarks is 225 s for fixed-point and 125 s for floating-point implementations, respectively. The median run-time for the successful benchmarks is 15.7 s for fixed-point and 17.8 s for floating-point implementations, respectively. We consider these times to be short enough to be of practical use to control engineers, and thus assert the success of the overall objective of the study that we have raised above. The completeness threshold depends upon the controller selected, and the SAT/SMT solver picks a controller with a low completeness threshold for all the time discretisations that we solve. The completeness threshold is zero where the controller synthesised results in a system with real valued eigenvalues.

A link to the full experimental environment, including scripts to reproduce the results, all benchmarks and the tool, is provided in the footnote as an Open Virtual Appliance (OVA).^2^ The provided experimental environment runs multiple discretisations for each benchmark, and lists the fastest as the resulting synthesis time.

In this article we have presented a selection of case studies for which we have been able to automatically synthesise safe controllers. In the full experimental environment we have included further case studies that we have been unable to synthesise controllers for. This is in many cases due to timeouts, especially when the set completeness threshold is too large, or when a controller simply may not exist for a benchmark at a given time discretisation and required controller precision. Yet another source of incompleteness is the inability of the synthesize phase to employ a large-enough precision for the plant model.

### Threats to generality

We identify the following factors as potential limits to the generality of the obtained results.

- *Benchmark selection:* We report an assessment of both our approaches over a diverse set of real-world benchmarks. Nevertheless, this set of benchmarks is limited within the scope of this paper and the performance may not generalize to other benchmarks.
- *Plant model precision and discretization heuristics:* Our algorithm to select suitable finite-precision arithmetic to model the plant behavior increases the precision by 8 bits at each step, in order to be compliant with the CBMC type API. Similarly, for time-discretization, we try a set of pre-defined time discretisations. This works sufficiently well for our benchmarks, but performance may suffer in some cases, for example if the completeness threshold is high.

## Conclusions

We have presented an automated approach to synthesize provably-correct digital state-feedback controllers that ensure safety over state-space models. Our approach is novel within the digital control literature: we provide a fully automated synthesis method that is algorithmically and numerically sound, considering various error sources in the implementation of the digital control algorithm and in the computational modeling of plant dynamics. Our experimental results show that we are able to synthesize automatically safe controllers for diverse benchmarks from the digital control literature, within a reasonable amount of time.

## Footnotes

1 https://github.com/johnmcfarlane/cnl.

2 https://www.cprover.org/DSSynth.
